# Administration of Cilostazol Prior to Intravenous Alteplase for Acute Branch Atheromatous Disease: A Report of Three Cases

**DOI:** 10.1155/crnm/3508206

**Published:** 2025-08-11

**Authors:** Eijirou Tanaka, Yoshinari Nagakane, Daiki Fukunaga, Daisuke Nakashima, Takehiro Yamada

**Affiliations:** Department of Neurology, Kyoto Second Red Cross Hospital, Kyoto, Japan

## Abstract

Patients with acute branch atheromatous disease often experience early neurological deterioration (END) in the first 24-h period, even after intravenous alteplase. Three cases treated with cilostazol, a phosphodiesterase 3-inhibitor, prior to intravenous alteplase with the aim of mitigating END are described. All three patients had no bleeding complications and good outcomes at 3 months, although two patients showed END within 24 h after intravenous alteplase.

## 1. Introduction

Branch atheromatous disease (BAD) is one of the stroke subtypes, distinguished from lacunar infarction by atheromatous occlusion at the orifice of one or a few penetrating arteries [1]. BAD has a higher incidence of early neurological deterioration (END) and poorer functional outcomes than lacunar infarction [2]. Intravenous alteplase has been reported to be effective enough in patients with lacunar infarction, as well as other clinical etiologies [3]. However, whether intravenous alteplase is also effective for BAD remains controversial. An observational study of intravenous alteplase for BAD patients reported that neurological symptoms often deteriorate after transient improvement within 24 h of intravenous alteplase [4]. Cilostazol, a phosphodiesterase 3-inhibitor, is an antiplatelet agent that has shown to prevent recurrent cerebral infarction, similar to aspirin [5], but with a lower risk of cerebral hemorrhage than aspirin, especially in subcortical infarction [6]. To prevent END and improve outcomes, using cilostazol just before intravenous alteplase for BAD seemed to be a good approach. No previous studies have examined the efficacy and safety of intravenous alteplase with preceding cilostazol, and therefore the feasibility of adding cilostazol 200 mg prior to intravenous alteplase in patients with BAD was evaluated. The present report describes the first three BAD cases treated with cilostazol prior to intravenous alteplase. This study was approved by our institutional review committee.

## 2. Case Presentation

### 2.1. Case 1

**Figure 1:**
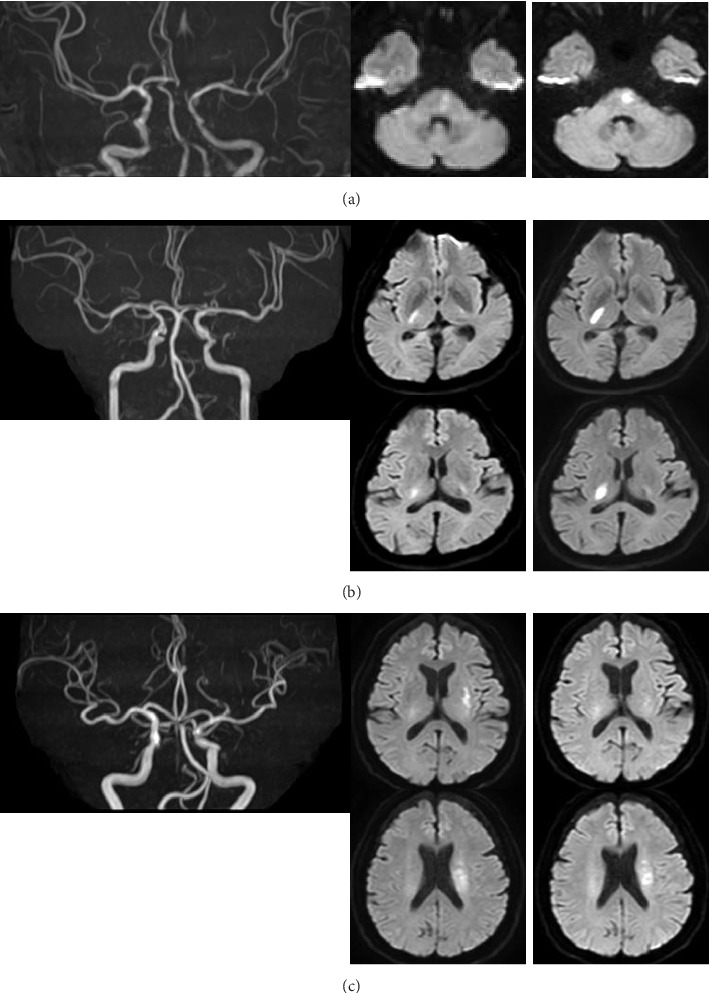
Cases of branch atheromatous disease with prior administration of cilostazol with intravenous alteplase. Images in Case 1 (a), Case 2 (b), and Case 3 (c). The index stroke is arranged on the left side (one MRA and one or two DWIs) and follow up on the right side. The follow-up images were taken at, respectively, Day 2 (Case 1), Day 3 (Case 2), and Day 11 (Case 3).

A 51-year-old man with hypertension, diabetes mellitus, and dyslipidemia presented with right hemiparesis and dysarthria that occurred 48 min before visiting our emergency department. On examination, the patient was alert and had a blood pressure of 170/98 mmHg, a regular heart rate of 113 beats/minute, and a temperature of 37.0°C. On neurological examination, right hemiparesis, and dysarthria were evident. The National Institutes of Health Stroke Scale (NIHSS) score was 9 points at the time of admission. Magnetic resonance imaging (MRI) showed an acute ischemic lesion at the left side of the pons, and magnetic resonance angiography (MRA) showed normal findings (Figure 1(a)). Immediately after obtaining consent from the patient, cilostazol 200 mg was administered prior to intravenous alteplase (0.6 mg/kg, onset to needle time [OTN]: 133 min). Although symptoms were improving to an NIHSS score of five points with intravenous alteplase, the right hemiparesis started to worsen again 30 min after completion of intravenous alteplase, resulting in an NIHSS score of nine points 6 h after intravenous alteplase. Multiple antithrombotic therapy, that is continuous infusion of argatroban 60 mg/day, cilostazol 200 mg/day, and clopidogrel 75 mg/day (with an initial loading dose of 300 mg), was started 24 h after intravenous alteplase, and no further neurological deterioration was observed. Continuous infusion of argatroban was administered for 4 days, and dual antiplatelet therapy was continued for 90 days. At 90 days, atrial fibrillation was detected, so direct oral anticoagulant monotherapy was selected. The follow-up MRI performed on Day 2 showed no infarct expansion. The NIHSS score remained at nine points on Day 7, and the modified Rankin Scale (mRS) score was 3 on Day 90. There were no hemorrhagic complications up to Day 90.

### 2.2. Case 2

A 67-year-old man with hypertension and a smoking habit of 15 cigarettes/day developed left hemiplegia 32 min before presenting to our emergency department. On examination, the patient was awake, with a blood pressure of 213/112 mmHg, a regular heart rate of 93 beats/minute, and a temperature of 36.1°C. On neurological examination, left hemiplegia was evident, with an NIHSS score of nine points at the time of admission. MRI showed an acute ischemic lesion at the right posterior limb of the internal capsule, and MRA showed no abnormal findings (Figure 1(b)). With the patient's consent, cilostazol 200 mg prior to intravenous alteplase was administered (0.6 mg/kg, OTN: 89 min). Immediately after the start of intravenous alteplase, the left hemiplegia improved to an NIHSS score of four points, but it then slightly worsened (NIHSS score of five points) within 12 h of intravenous alteplase. Multiple antithrombotic therapy, that is continuous infusion of argatroban 60 mg/day, cilostazol 200 mg/day, and clopidogrel 75 mg/day (with an initial loading dose of 300 mg), was started 24 h after intravenous alteplase, and no further neurological deterioration was observed. Continuous infusion of argatroban was administered for 4 days, and dual antiplatelet therapy continued for 90 days. We followed cilostazol as a single antiplatelet therapy. The follow-up MRI performed on Day 3 showed infarct expansion at the right posterior limb of the internal capsule. The NIHSS score was 3 points on Day 7, and the mRS score was 3 on Day 90. There were no hemorrhagic complications up to Day 90.

### 2.3. Case 3

An 82-year-old man with hypertension presented with right hemiplegia and severe dysarthria that occurred 211 min before visiting our emergency department. On examination, the patient was alert and had a blood pressure of 168/82 mmHg, a regular heart rate of 84 beats/minute, and a temperature of 36.3°C. On neurological examination, right hemiparesis and dysarthria were evident, with an NIHSS score of seven points at the time of admission. MRI showed an acute ischemic lesion at the left corona radiata and putamen, and the findings of MRA were normal (Figure 1(c)). Immediately after acquiring consent from the patient, cilostazol 200 mg prior to intravenous alteplase was administered (0.6 mg/kg, OTN: 265 min). The symptoms improved to an NIHSS score of six points within 24 h of intravenous alteplase, and then multiple antithrombotic therapy, that is a continuous infusion of argatroban 60 mg/day, cilostazol 200 mg/day, and clopidogrel 75 mg/day (with an initial loading dose of 300 mg), was administered. No neurological deterioration was observed during and after intravenous alteplase. Continuous infusion of argatroban was administered for 4 days, and dual antiplatelet therapy continued for 90 days. We followed clopidogrel as a single antiplatelet therapy. The follow-up MRI performed on Day 11 showed no infarct expansion. The NIHSS score was 3 points on Day 7, and the mRS score was 2 on Day 90. There were no hemorrhagic complications up to Day 90.

## 3. Discussion

In the NINDS trial, intravenous alteplase was reported to show a similar positive effect in all stroke subgroups, including lacunar infarction [7]. Wu et al. reported that patients with BAD also had better outcomes and fewer neurological symptoms in the intravenous alteplase group than in the nonintravenous alteplase group in a retrospective, case-control study with propensity score matching [8]. However, END is an important clinical issue for patients with BAD who experience neurological improvement after intravenous alteplase. Park et al. reported that six of nine patients in the intravenous alteplase group had END after neurological improvement [9]. A similar phenomenon was reported by Deguchi et al. in which 6 of 8 BAD patients had neurological improvement after intravenous alteplase, but four of them had END within 24 h [4]. END including progressive motor deficit may cause residual functional disability.

The standard strategy for BAD in the absence of intravenous alteplase is antiplatelet agents, which may also be applied to END [10]. On the premise that intravenous alteplase alone was considered insufficiently effective for BAD patients, we adopted the strategy of administering antiplatelet agents prior to intravenous alteplase to prevent END, especially for the first 24 h after intravenous alteplase, when postintravenous alteplase antithrombotic treatment is not recommended. Several reports have shown the effect of intravenous alteplase with antiplatelet agents, with or without preonset medications [11–13]. There was no significant difference in functional outcome and no increase in symptomatic intracranial hemorrhage in stroke subjects, either with single or dual antiplatelet therapy. The ARTIS study, in which aspirin 300 mg was added to intravenous alteplase, showed improvement of functional outcome at 3 months but an increase in intracranial hemorrhage [14]. Cilostazol is supposed to have a lower risk of bleeding and an equal effect in preventing stroke compared with aspirin, and therefore, we chose cilostazol as the antiplatelet agent prior to intravenous alteplase [5, 6].

The most important result of the present study was the absence of bleeding complications in the three patients treated with cilostazol prior to intravenous alteplase. Although this was a finding in only three patients, there was no discernible increase in bleeding risk. As for motor paralysis, all 3 patients showed neurological improvement immediately after administration of intravenous alteplase, but 2 (66.7%) worsened again within 24 h. At 3 months, the functional outcome was an mRS score of 3 in two patients who experienced clinical worsening and an mRS score of 2 in one patient who did not worsen. These current study outcomes were comparable to those of previous studies without antiplatelet therapy preceding intravenous alteplase, and the effect of additional cilostazol was not obvious because only three cases were examined. While we selected cilostazol, an antiplatelet agent considered to have a lower bleeding risk, our study results demonstrated no apparent efficacy. The weak antithrombotic effect of cilostazol may have contributed to the lack of efficacy, but given the small sample size of three cases, further studies using a larger number of BAD patients with cilostazol prior to intravenous alteplase are warranted. Recently, there have been reports on the treatment strategy of alteplase plus argatroban [15]. Compared to the control group receiving alteplase alone, there were no differences in efficacy or safety, but the study population included all stroke patients. Essentially, efficacy should be compared in BAD patients, such as those in the present study, and such treatment strategies also should be considered in the future.

In two cases (Case 1 and Case 2) where END occurred, inconsistencies were observed between symptoms and imaging findings following intravenous alteplase. As noted, Case 1 showed no significant expansion of DWI high signal intensity following END, whereas Case 2 demonstrated obvious expansion of DWI high signal intensity. Despite the expansion of DWI high signal, Case 2 showed neurological improvement from Day 3. To understand these gaps, it is essential to recognize that during the acute phase of a cerebral infarction, there is heterogeneity in the severity of ischemia within the regions exposed to ischemia in the territory of the occluded vessel. The areas with DWI high signal intensity represent irreversible tissue damage due to severe ischemia, while the remaining areas may become reversible upon reperfusion. As a result, there may be a gap between neurological severity and the volume of DWI high signal intensity. In Case 1, the entire area perfused by the occluded perforating branch appeared as DWI high signal intensity on the initial MRI, but there were areas of faint DWI signal intensity changes within it. On the other hand, in Case 2, the area of perfusion in the occluded perforating branch was relatively wide, and severe neurological deficits were present; however, limited areas showed DWI high signal intensity consistent with infarction.

## 4. Conclusion

In conclusion, there were no bleeding complications in three patients with BAD who received cilostazol prior to intravenous alteplase. All had symptom improvement immediately after intravenous alteplase, and they recovered to walking independence 3 months later, although two patients showed END within 24 h after intravenous alteplase.

## Data Availability

The data that support the findings of this study are available from the corresponding author upon reasonable request.
